# Immunomodulatory Effects of Glutathione, Garlic Derivatives, and Hydrogen Sulfide

**DOI:** 10.3390/nu11020295

**Published:** 2019-01-30

**Authors:** Camila Rodrigues, Susan S. Percival

**Affiliations:** Food Science & Human Nutrition Department, University of Florida, Gainesville, FL 32611, USA; crodrigues@ufl.edu

**Keywords:** aged garlic extract, glutathione, hydrogen sulfide, immune response, inflammation

## Abstract

Glutathione and aged garlic extract are sulfur-containing products that play important protective and regulatory roles within the immune system and in oxidative processes. Hydrogen sulfide (H_2_S), an endogenous, gaseous, signaling transmitter, has also been shown to be involved in the regulation of inflammation. Recent studies have shown that sulfur-containing compounds from garlic have beneficial effects in attenuating outcomes associated with cardiovascular disease and inflammation by a mechanism that may be related to the H_2_S signaling pathway. In this review, we summarize the main functions of glutathione (GSH), garlic derivatives and H_2_S and their role in the immune response and impact on health and disease.

## 1. Introduction

Glutathione (GSH) is required by the immune system for two important reasons: it protects host immune cells through its antioxidant mechanism and it provides the optimal functioning of lymphocytes and other cells of the immune system [1]. Conditions under which GSH supplementation may be recommended occur in the elderly who have low levels of GSH and in individuals with chronic inflammation [2]. Garlic derivatives and aged garlic extract (AGE), an odorless product containing S-allylcysteine (SAC) and S-allylmercaptocysteine (SAMC), have shown immunomodulatory effects by improving the immune response, resulting in attenuation of the effects of cardiovascular disease and inflammatory-associated processes [3,4]. In addition, others have demonstrated that sulfur compounds from AGE play an important regulatory role in the inflammatory response through the prevention of NF-kB activation, which leads to the suppression of the production of pro-inflammatory cytokines such as TNF-α, IL-6, and IL-1β [5,6].

Hydrogen sulfide (H_2_S) was discovered to be endogenously produced by cystathionine beta synthetase (CBS), cystathionine gamm-lyase (CSE) and later by 3-mercaptopyruvate transferase (3-MST) under physiological conditions [7,8]. More recently, it has been suggested that H_2_S be used as a targeted therapy for diseases like cancer, Alzheimer’s disease, atherosclerosis and hypertension [9,10,11]. Low levels of H_2_S have been associated with the inward hypertrophic remodeling of the microvasculature involved in obesity, while its replenishment aids in the attenuation of this process [12]. H_2_S also plays an important role in inflammation by regulating the post-translational modification of the NF-κB pathway. However, several studies have shown that H_2_S can act in both ways by activating and inhibiting the activation of NF-κB, which may depend on experimental conditions as well as the concentration of the exogenous donor [13,14].

The objective of this review is to summarize the main functions of GSH, garlic derivatives and H_2_S and their role in the immune response and impact on health and disease.

## 2. Glutathione

Reduced glutathione (GSH) is a tripeptide (glutamate-cysteine-glycine) found in plants and animals, which acts as a major hydrophilic, intracellular antioxidant, protecting cells against endogenous and exogenous toxins including reactive oxygen species (ROS) and radical nitrogen species (RNS). GSH acts as a nucleophile and as a reducing agent by reacting with and eliminating electrophilic or oxidizing species, thus preventing the damage of protein, lipid and nucleic acid molecules [15].

GSH can be obtained through the diet by consuming foods like meat, fish, broccoli, cabbage, garlic, onions, cereals, dairy products and dietary supplements [16,17]. However, this has a miniscule contribution to whole body GSH, since dietary GSH can be hydrolyzed by γ-glutamyltranspeptidase (GGT) in the gastrointestinal tract and it has inefficient cellular transport, hence its bioavailability is low [18]. In contrast, GSH can be synthesized by the human body in large amounts, especially in the liver, as a consequence of two ATP-dependent enzymatic reactions by precursor amino acids cysteine, glutamate and glycine in the trans-sulfuration pathway. The homocysteine/methionine cycle provides a substrate that undergoes the trans-sulfuration pathway to produce GSH. This pathway is mainly controlled by cystathionine β-synthase (CBS) and cystathionine γ-lyase (CSE), two enzymes needed for L-cysteine biosynthesis, both of which require vitamin B6 and are important for maintaining normal levels of GSH in the system [19,20].

The first step of GSH synthesis is controlled by glutamate-cysteine ligase (GCL), which is ATP-dependent and catalyzes a reaction to form γ-glutamylcysteine. This enzyme is a rate-limiting step in the pathway and can be allosterically regulated by feedback inhibition, dependent on GSH and L-cysteine availability. The second step, in which glycine incorporates with γ-glutamylcysteine to form GSH, is catalyzed by glutathione synthetase (GSS) [21]. Although GSS is not rate-limiting in glutathione synthesis, some studies have revealed that GSS and GCL are coordinately regulated and may play a role in overall GSH synthesis capacity in specific tissues or under trauma conditions [22,23,24]. Furthermore, GSH can be metabolized by gamma-glutamyl transferase (GGT) to form glutamate and cysteine-glycine in the γ-glutamyl cycle, by transferring a glutamyl moiety to a series of acceptor molecules such as α-amino acids. This mechanism helps to maintain the intracellular levels of cysteine [25,26].

GSH plays a role as a potent antioxidant in both plants and animals, preventing cellular damage caused by ROS that are being continuously produced by cells, either as the result of electron transfer or as products of enzymatic reactions responsible for cell oxidative and degenerative processes. The process is mediated by GPx, which oxidizes GSH in glutathione disulfide (GSSG), the oxidized form of GSH, and reduces free H_2_O_2_ to water and lipid hydroperoxide to stable lipid alcohol. GSSG is further reduced by glutathione reductase (GR) and is dependent on NADPH to regenerate GSH and start the process all over again. GSH can also react non-enzymatically with O_2_^•−^, NO, ^•^OH and ONOO^−^, and neutralize these free radicals [27]. This tripeptide is mainly present in the cytosol in a specific concentration range (~1–10 mM), and kept in a regulated amount within the mitochondria, endoplasmic reticulum (ER), nuclear matrix and peroxisomes. Plasma GSH is usually found in low concentrations (~10–25 µM) because it can be rapidly oxidized in a non-enzymatic reaction to GSSG in the presence of ROS/RNS [28,29,30]. These concentration values will vary according to the type of cell and the compartment that is being analyzed, such as plasma, intercellular or extracellular.

It is known that cysteine, GSH, and H_2_S are closely linked in metabolic processes. There are factors that may contribute to an increase in GSH levels when H_2_S levels rise [31]. H_2_S has been described as playing a role in changing intracellular levels of GSH by increasing the transport of cysteine, helping the redistribution of GSH into mitochondria and contributing to the protection of cells from oxidative stress [32]. H_2_S has also been shown to act as a cytoprotective molecule against oxidative stress in SH-SY5Y cells undergoing HOCl-induced cytotoxicity, which is comparable to the effect of GSH [33]. In addition, enzymes required for the conversion of methionine into cysteine have been shown to decline with age, affecting GSH synthesis and the consequent reduction of H_2_S levels in the body [34].

Considering all the beneficial effects of GSH in the immune response, consuming food that can increase GSH levels is a good alternative to GSH dietary supplementation, since GSH is known to have low bioavailability. The intake of sulfur-rich food is a good example of how to increase endogenous GSH levels, since an excess of cysteine and methionine can be stored in the body in the form of GSH or be metabolized and excreted [35]. Chen et al. tested the effect of cruciferous vegetables on colonic GSH levels. In this study, male rats with an induced colon tumor were given a diet supplemented with 10–40% lyophilized cabbage and broccoli for 14 days and levels of colon mucosal GSH were measured. Results demonstrated that GSH levels in the colon significantly increased in the treatment group while in a parallel group, oral GSH supplementation did not affect endogenous GSH levels [36]. In a cell culture study, supplementation with sulforaphane, a cruciferous vegetables inducer, significantly increased total levels of GSH by around 106% and also increased GR (~20%) and GST (~130%) activities in human neuroblastoma cells [37]. In previous studies, coffee intake has been shown to affect GSH levels. Esposito et al. demonstrated that an intake of 5 cups of coffee per day for 7 days increased plasma GSH levels by 16% in healthy individuals without an increase in homocysteine levels [38]. In a similar approach, authors supplemented healthy male individuals with 5 mg/kg/day of pure caffeine (equivalent to approximately 5 cups/day) for 7 days. Data showed an increase of 106% on GSH levels and a reduction of 70% on GSSG levels, which elevated the GSH/GSSG ratio to 249% [39]. 

## 3. Garlic (*Allium sativum* L.)

Garlic (*Allium sativum* L.) is a member of the *Liliaceae* family and its consumption has been dated back over 6000 years as a food ingredient with medicinal properties. Garlic contains organosulfur compounds that provide a unique odor and flavor, and potential health benefits [40]. The major organosulfur compounds found in fresh garlic are S-allyl-L-cysteine sulfoxides (alliin) and γ-glutamyl-S-allyl-L-cysteines (sulfoxides). Once it is crushed or cut, these compounds are enzymatically converted into alliin and subsequently to allicin, a highly unstable thiosulfinate. Thereafter, allicin is rapidly decomposed to organosulfur volatiles: diallyl trisulfide (DTS), diallyl disulfide (DADS), diallyl sulfide (DAS) and sulfur dioxide (SO_2_) [41,42]. Contrarily, the process of aqueous extractions rapidly converts γ-glutamyl-S-allylcysteine to SAC, a water-soluble compound [43]. 

Different types of garlic supplements are commercially available, and the profile of sulfur-containing compounds varies according to manufacturing processes. Several in vivo and in vitro studies have demonstrated the beneficial effects of garlic consumption, such as antioxidant [44,45,46], cardioprotective [47], anti-inflammatory [46,48] and anti-microbial [49], and these effects are mediated through different mechanisms, depending on the sulfur-containing compound profiling. Among the different supplements, aged garlic extract (AGE) accounts for the most studied product associated with health benefits.

### Aged Garlic Extract (AGE)

AGE is a product of the prolonged extraction of fresh garlic soaked in aqueous/ethanol solution at room temperature for 20 months, which is commonly known as an aging process. This process results in an odorless product containing a variety of water-soluble allyl amino acid derivatives, especially SAC and S-allylmercaptocysteine (SAMC), which comprise the majority of the organosulfur compounds, and minor amounts of oil-soluble organosulfur volatiles in addition to other flavonoids and saponins [50]. These major compounds are present in the extract due to its aging process unlike fresh garlic, which is high in allicin and alliin. Due to its high bioavailability, SAC is easily absorbed in the gastrointestinal tract and distributed among different organs where it exerts a protective effect against oxidative processes within and around the cells [51]. In a human pharmacokinetic study, healthy volunteers were given a single dose of 500 mg of AGE in order to measure the levels of SAC in the blood. In this study, SAC was found in the blood after 1 h of oral administration of AGE, and traces were found even after 10 h after consumption [52]. 

The antioxidant activity is the most known and explored property of AGE and it is related to the presence of the water-soluble organosulfur compounds. In fact, it has been suggested that AGE has more potent antioxidant properties than fresh garlic extract [53]. Constituents of AGE have the ability to scavenge ROS [54,55] and suppress their generation [56], consequently reducing the oxidative damage and mitigating the effect of aging [57]. Recently, AGE has been suggested to alleviate metabolic syndrome-induced cardiovascular risk in rats through its antioxidant property [58].

AGE has also been reported to reduce oxidative stress via activation of the Nrf2-ARE pathway [59,60]. Likewise, AGE has been demonstrated to attenuate the effects of cardiovascular disease by lowering blood cholesterol, triglycerides, blood pressure and inhibiting blood coagulation [61,62]; reduce diabetes [63] and obesity [4]; and prevent diverse types of cancer [64,65] and neurodegenerative disorders such as Alzheimer’s disease [66]. 

## 4. Hydrogen Sulfide

Hydrogen sulfide (H_2_S) is a colorless and flammable toxic gas with a characteristic smell of rotten egg and it can be found in natural gas, volcanic emissions, petroleum and decomposition of organic matter. H_2_S is believed to originate from hydrothermal vents along the separation lines of tectonic plates. Recent theories have raised the question that life originated from the heat generated by the process of hydrothermal circulation that provides energy mainly from H_2_S [67]. This gas is a small lipophilic molecule that can penetrate cell membranes without transporters and can cause brain and pulmonary damage when in high concentrations. High oxygen concentration can also increase toxicity of high H_2_S levels leading to vasoconstriction and inhibition of cellular proliferation [68,69,70]. Additionally, acute exposure to high H_2_S concentrations has been shown to cause neurodegenerative damage in mice [71]. 

In 1989, Warenycia and Goodwin [7] first demonstrated the presence of endogenous H_2_S in the human brain, also showing a selective uptake of exogenous H_2_S in the brainstem. H_2_S was discovered to be an endogenous gas produced by two cytosolic enzymes: CBS and CSE. These two enzymes, as mentioned before, are rate limiting steps on the trans-sulfuration pathway having GSH as a final product, and both catalyze multiple H_2_S-generating reactions utilizing cysteine and homocysteine as substrate. The regulation of this mechanism is highly dependent on substrate availability. A third enzymatic pathway that metabolizes H_2_S is mediated by cysteine aminotransferase (CAT) and 3-mercaptopyruvate sulfurtransferase (3-MST) in the cysteine catabolism pathway [8,72,73]. These enzymes are expressed in different tissues and also in different cell compartments. CBS and CSE are responsible for metabolizing cysteine and/or homocysteine to produce H_2_S along with other metabolites such as homolanthionine and cystathionine; whereas 3-MST catabolizes 3-mercaptopyruvate to pyruvate, simultaneously releasing H_2_S in a process that catalyzes the transfer of sulfane sulfur from persulfides or thiosulfates to an acceptor [74,75,76]. 

Over the past 25 years, the H_2_S concept has gone from a toxic gas to a signaling molecule with potential clinical relevance. Hence, H_2_S has become the third endogenous gaseous signaling transmitter among nitric oxide (NO) and carbon monoxide (CO) [69,77]. Recently, H_2_S has been described as a target molecule for therapies for specific diseases such as cancer, Alzheimer’s, atherosclerosis and hypertension [9,10,78,79]. Studies have demonstrated that H_2_S plays a role in a variety of pathways, regulating important mechanisms in our body as a signaling molecule. H_2_S interacts with a variety of ion channels and receptors, such as K_ATP_, Ca^2+^, Cl^−^ channels and TRVP1 and TRPA1 receptors, modulating different responses [80]. Moreover, H_2_S behaves as a cardioprotective molecule involved in different mechanisms [81,82]. 

It is known that H_2_S regulates the Keap1-Nrf2 pathway, the major regulatory cytoprotective response against oxidative stress, resulting in an increased expression of AREs. In an animal study, H_2_S activates Nrf2 pathway through modification in Keap1 protein, leading to activation of several ARE-driven genes that include *Cbs*, *Cse* and *Sqrdl.* H_2_S has been shown to stabilize Nrf2 through inhibition of Keap1 in mouse embryonic fibroblasts. The same study demonstrated that H_2_S upregulates the expression of *Cbs* in a Nrf2-dependent manner [83]. One mechanism proposed is through S-sulfhydration of Keap1 leading to its dissociation from Nrf2, consequently enhancing Nrf2 nuclear translocation and further activation of ARE genes. This was confirmed by increased GSH production [84,85]. S-sulfhydration is a post-translational modification of protein cysteine residues that can either activate or inactive a signaling pathway [86]. 

H_2_S has also presented the ability to inhibit the activity of phosphodiesterases (PDEs), which is involved in vascular smooth muscle relaxation, leading to vasodilation and reducing blood pressure [87]. Moreover, this gasotransmitter has been described as related to gastric ulcers, acting as a promoter healing, which is produced by CSE and CBS in the gastric mucosa in response to injury, whereas inhibition of H_2_S can contribute to gastric injury caused by anti-inflammatory drugs [88,89]. Recently, lower levels of H_2_S have been associated with microvascular inward hypertrophic remodeling involved in obesity, while its replenishment helps to attenuate this whole process [12]. 

H_2_S is considered to be a toxic gas and its administration is considered to be dangerous. Previous data have demonstrated that some food and dietary approaches may boost endogenous H_2_S levels in a physiological rate and bring the beneficial effects cited above. Constituents of fresh garlic and AGE, as mentioned in this review, have the potential to increase endogenous H_2_S. Benavides et al. [90] showed that garlic-derived organic polysulfides treatment was able to increase H_2_S levels in red blood cells, and consequently, modulate vasorelaxation is smooth muscle cells. Likewise, Predmore et al. [91] demonstrated that DATS from fresh garlic had the ability to increase H_2_S levels in blood and myocardial tissues in mice. Dietary restriction is one alterative that is known to affect endogenous H_2_S production. In fact, Hine et al. [92] suggested that sulfur amino acid restriction can contribute to increased longevity and stress resistance by increasing the expression of CSE, resulting in the increase of H_2_S production in a mouse model study. In another study, Wang et al. [93] demonstrated that 30% of dietary restriction was able to increase the expression of CSE and CBS, and endogenous H_2_S levels in aged rats, consequently, reducing the level of oxidative stress and renal aging. 

## 5. Current Status of Knowledge

### 5.1. Glutathione and Immunity

GSH is involved in several other cellular events, such as synthesis of proteins and DNA, transport, enzyme activity, nutrient metabolism, cell protection and protein S-glutathionylation [94,95]. Moreover, GSH is essential for the regeneration of other antioxidants, like vitamin E and C [15]. GSH also participates in a series of immune processes, protecting host immune cells through its antioxidant mechanism and providing the optimal functioning of lymphocytes and other cells of the immune system. Endogenous GSH is essential for T-cell proliferation, dendritic cell functions, in which authors demonstrated that cysteine supplementation mediates the redox modeling of Tregs through the reduction of GSH synthesis in isolated dendritic cells from mice. In addition, they found that during T cell activation, GSH transport from the nucleus to the cytoplasm was blocked [96]. GSH is also important for the activity of polymorphonuclear neutrophils (PMN). It was observed that when isolated leukocytes from healthy humans were treated with GSH-oxidizing reagents, phagocytosis was regulated in PMN through the inhibition of the assembly of microtubules and consequent reduction of H_2_O_2_ production in GSH homeostasis [97]. 

The immune system is highly affected by the levels of GSH in the body and even small changes in intracellular GSH levels can affect lymphocyte activity [1]. Hadzic et al. [98] have demonstrated that when depleting GSH from T cells isolated from mice, T cell proliferation and IL-2 production were reduced. Likewise, when GSH levels were restored with N-acetycysteine (NAC), a precursor of L-cysteine, supplementation, T cell proliferation and IL-2 levels were increased. More recently, it has been suggested that GSH supplementation may protect cells from immunological cell damage by inhibiting the complement activation cascade and the binding of antibodies to antigens in isolated glomerular mesangial cells from rats, suggesting the possible use of GSH in the treatment of certain immune disorders [99].

Human clinical studies have revealed that GSH and cysteine levels are decreased in HIV-infected patients, leading to impairment in the functioning of the immune system. Supplementation with NAC (~7 g/day) has been shown to increase the levels of intracellular GSH during HIV infection, improving the immune response and increasing protection against oxidative stress caused by low levels of GSH in those patients [100]. In a recent double-blind study in a group of HIV-infected individuals with low CD4+ cell counts, 3 months of GSH supplementation (1.26 g/day) restored levels of intracellular GSH, consequently alleviating oxidative stress and balancing the production of cytokines by increasing the production of IL-10 and decreasing the levels of IL-12, IL-2, and IFN-γ [101]. Pena et al. [102] investigated the effect of a GSH precursor supplementation in patients with cirrhosis. In their study, subjects receiving intravenous GSH prodrug at a dose of 70 mg/kg 3 times a day for 8 days had a reduction in TNF-α, IL-8 and IL-6 levels. Another study revealed that even moderate GSH depletion is able to downregulate TNF-α dependent on NF-kB activation in cultured hepatocytes, with a major role in GSH/GSSG redox status [103]. Previous data for glutathione and its effects in the immune response are summarized in Table 1.

Nrf2-ARE is the sole pathway that controls the enzymes responsible for GSH production, such as GCL and it also helps to support GSH utilization through several regulating mechanisms. Compounds that can activate the Nrf2-ARE pathway are called Nrf2 activators, such as R-α-lipoic acid, sulforaphane and S-allylcysteine (SAC) from garlic. These compounds can increase the activation of this pathway, consequently leading to increased production of antioxidant products [104,105]. Likewise, the inhibition of Nrf2 response has been shown to cause an accumulation of GSSG and increased cytotoxicity in an animal model study, which indicates that Nrf2 is critical for the maintenance of GSH homeostasis, a mechanism that is important to minimize the effects of oxidative stress and boost the immune response [106]. 

Several conditions can affect the levels of intracellular GSH, such as smoking, excessive alcohol use, drugs, UV radiation, obesity, age, type 2 diabetes and cardiovascular diseases [107,108,109,110,111,112]. In addition, conditions in which amounts of GSH may be required under appropriate circumstances also occur in elderly people [2,113,114], in individuals infected with tuberculosis [115,116], HIV [117,118], in episodes of sepsis or shock [119] and inflammatory conditions such as colds and flu [120]. In consequence, a decrease in GSH levels can have adverse effects in diverse physiological processes. Studies have demonstrated that immune functions are impaired under low conditions of GSH, such as T cells proliferation and NK activity [115,121]. In situations where GSH synthesis was inhibited by L-buthionine-sulfoximine (BSO), an inhibitor of GCL, DNA synthesis in naïve human CD4+ T cells was impaired, suggesting that T cells require GSH for normal proliferation [122]. 

### 5.2. Fresh Garlic Derivatives and Immunity

The immunomodulatory effect of fresh garlic derivatives is evidenced in a variety of studies and in different physiological states. Hung et al. [123] reported that DATS supplementation (1 and 10 mg/kg/day) for 2 weeks promoted the activity of phagocytosis on PBMC from mice with leukemia. It has been documented that purified protein fraction from garlic extract modulates immunity in mice with implanted breast tumors. Results showed that animals treated with different doses of garlic fractions had increased T-cell activation and an increase of intra-tumor infiltration of lymphocytes in all doses [124]. Hassan et al. [125] reported that administration of 20 mg/kg of an isolated fraction of garlic extract caused a significant increase in NK activity in mice. The immunomodulatory effect of fresh garlic compounds has also been evidenced in humans. In a randomized crossover study, the consumption of a single meal containing 5 g of raw crushed garlic increased the gene expression of nuclear factor of activated T cell activating protein with immunoreceptor tyrosine-based activation motif 1 (*NFAM1*), a protein that is expressed in B cells, T cells, monocytes, which is involved with B cell development and signaling [126].

Fresh garlic constituents also play an important role in inflammation. In a cell culture model using isolated PBMC from healthy individuals, treatment with fresh garlic extract was able to decrease the expression of the pro-inflammatory cytokine, IL-17, in a dose dependent way when stimulated with PHA [48]. Quintero-Fabian et al. [127] investigated the effects of alliin in LPS-stimulated 3T3-L1 adipocytes. Cells incubated with 100 μmol/L of alliin for 24 h followed by LPS stimulation for 1 h, prevented the increase of IL-6, MCP-1 gene expression and protein levels. Authors also identified the up-regulation of an array of genes involved with immune response and a down-regulation of genes involved with tumor development. Allicin supplementation has been shown to protect against alcoholic fatty liver disease by improving inflammatory conditions and antioxidant functions. Results demonstrated that mice with alcoholic fatty liver disease receiving allicin supplementation in two different doses (5 mg/kg/day and 20 mg/kg/day) had a reduction in TNF-α, IL-1β and IL-6 with the 20 mg dose, and a reduction in several biomarkers of oxidative stress [46]. Human colorectal cancer cells supplemented with DADS (2.5–40 μmol/L) showed a decreased phosphorylation and nuclear translocation of NF-κB/p65 protein in a dose dependent way [128]. Recently, Shi et al. [129] demonstrated that oral administration of alliin (500 mg/kg) suppressed LPS-induced AP-1/NF-κB/STAT-1 activation in mice with colitis.

### 5.3. AGE and Immunity

Several studies have shown the effectiveness of AGE on modulating the immune response, which contributes to its protective effects (summarized in Table 2). In a cell culture model, AGE supplementation was able to increase NK cell activation and cytotoxic T cells from isolated spleen cells from mice. Results also showed increased levels of Il-2, TNF-α and IFN-γ [130]. In addition, AGE has been suggested as a preventive therapy for cancer through its immunomodulatory effects. In a mice study, AGE was able to modify cytokines production into a protective pattern by decreasing the levels of IFN-γ and IL-4 and reducing the number of T regulatory lymphocytes in the spleen [131]. Similarly, mice with implanted fibrosarcoma tumor receiving AGE over 28 days presented induced effective immune responses by increasing IFN-γ levels and both CD4+ and CD8+ T cells when compared to control group, which contributed to significant inhibitory effect on tumor growth and increased the life spans of the mice [132]. Nillert et al. [133] examined the neuroimmunomodulatory effect of AGE on β-amyloid-42-induced cognitive dysfunction in rats. The authors concluded that AGE significantly attenuated neuroinflammation by reducing IL-1β and the activation of microglia. These results indicate that AGE may be useful for reducing neural damage commonly associated with neurological disorders. 

The immunomodulatory effect of AGE is also evidenced in human studies where daily administration of AGE (500 mg/day) for 6 months led to an increase in the number of NK cells in patients with advanced colon, liver and pancreatic cancer. This result suggests that AGE confers a protective effect against death due to cancer, considering that control group patients who died rapidly showed a significant decrease in NK cell activity [64]. In addition, supplementation with AGE (2.56 g/day) for 45 days helped to improve immune response by increasing the proliferation of γδ-T cells and NK cells and attenuating the severity of reported symptoms during the cold and flu season in healthy adults [134]. Recently, daily consumption of AGE (3.6 g/day) modified immunity and inflammation by increasing γδ-T cell population and decreasing NKT cell population and IL-6 and TNF-α levels in the serum of adults with obesity [135]. 

Sulfur compounds present in AGE, especially SAC, have been proposed as a key regulator of the inflammatory response, mainly acting by attenuating NF-kB activation, consequently suppressing the production of pro-inflammatory cytokines such as TNF-α, IL-6, and IL-1β. In a cell culture model, Geng et al. [3] showed that Jurkat T cells treated with SAC in different doses was able to inhibit the NF-κB p50/65 heterodimer activation. Effects are also evidenced in animal studies. In a study using diabetic rats, oral administration of SAC significantly reduced the protein levels of NF-κB, TNF-α and TLR4 in the hippocampus, which can contribute to minimize the severity of neuroinflammation, consequently, attenuating neuronal injury [6]. Likewise, diabetic mice receiving SAC supplementation by drinking water had a reduction in renal levels of IL-6 and TNF-α and suppression of renal NF-κB activity, mRNA expression and protein production, a defensive mechanism that contributes to minimize kidney injury caused by diabetes [5]. Anandasadagopan et al. [136] have recently demonstrated that administration of SAC in rats significantly decreased the expression of NF-κB p65, TNF-α and inducible nitric oxide synthase (iNOS), exerting a protective effect against chromium-induced toxicity. Likewise, AGE has been shown to play an important role in the regulation of the Nrf2-ARE pathway. Hiramatsu et al. found that AGE promoted Nrf2 activation in a dose dependent manner using human endothelial cells, consequently increasing the expression of CGLC and HO-1 [59]. In particular, SAC has also shown potential effects on activating Nrf2 factor in neural cells, a mechanism that may contribute to its cytoprotective effects caused by chronic inflammation and oxidative stress [60]. 

### 5.4. Hydrogen Sulfide and Inflammation

In addition to other physiological effects, H_2_S also plays an important regulatory role in inflammatory and anti-inflammatory processes. H_2_S has been shown to reduce edema formation by inhibiting leukocyte-endothelial cell adhesion, to promote neutrophil apoptosis and to stimulate macrophages differentiation to an anti-inflammatory phenotype, which contributes to inhibiting the development of atherosclerosis [137,138,139]. A recent study has shown compromised H_2_S biosynthesis in obese microvasculature driven by macrophages from obese mice [140]. 

Additionally, H_2_S appears to play an important role in the modulation of the immune response by regulating the post-translational modification of the NF-κB (summarized in Table 3). Several studies have demonstrated that the role of H_2_S in NF-κB activation is very controversial, which may depend specifically on the levels of H_2_S. In a cell culture model, Huang and colleagues [14] demonstrated that exogenous H_2_S reduced the secretion of TNF-a, IL-6, PGE2, NO levels and expression of IL-1b, COX2, and iNOS in RAW64.7 cells treated with an H_2_S-donor (200 μM), as well as the inhibition of NF-κB activation. Similarly, pre-treatment with NaHS (0.01, 0.1 and 0.5 or 1 mM) significantly reduced TNF-α and IL-6 secretion in THP-1 macrophages following LPS stimulation, however, the mechanism associated with the anti-inflammatory response was said to be involved in epigenetics alterations in histones [141]. In an animal study, H_2_S-releasing agent was as shown to reduce expression of pro-inflammatory cytokines as a consequence of the inhibition of NF-κB activation. Authors have also observed the reduction of mRNA expression of TNF-α, IFN-γ, IL-1, IL-2, IL-12 levels in mice with colitis [142]. Similarly, administration of exogenous H_2_S significantly reduced TNF-α expression and protein level, which consequently reduced the severity of colitis in rats. Inhibition of H_2_S led to significant intestinal inflammation in those animals, which can contribute to alleviating the severity of the disease [143]. H_2_S has also been shown to reduce airway inflammation in an LPS-induced model in mice through the prevention of neutrophil increase in bronchoalveolar lavage fluid as well as reduction of IL-1β levels [144]. Recently, treatment with H_2_S decreased the renal expression of TNF-α, IL-6, IL-10 levels as well the activation of the NF-kB pathway, and increased the activities of GPx and superoxide dismutase in rats with chronic renal failure [145]. 

In contrast, a series of studies have reported that H_2_S mediates the activation of NF-kB. Zhi and colleagues [13] have suggested that exogenous H_2_S (0.01, 0.1 or 1 mM) in higher concentration than the physiological range was able to induce the synthesis of TNF-α, IL-1β, and IL-6 by activation of NF-κB in cultured THP-1 cells. They also proposed a mechanism in which this signaling molecule mediates inflammatory activity through the activation of mitogen-activated protein kinase 1 (MEK1), extracellular signal–regulated kinases (ERK1/2) phosphorylation, resulting in IkBα degradation and subsequently increasing NF-κB-binding activity. Another study has demonstrated that H_2_S was able to mediate NF-κB antiapoptotic activity under inflammatory stimulation in liver cells. In this study, TNF-α was used to stimulate the transcription of CSE generating H_2_S [146]. A more recent research conducted by Badiei and colleagues [147] using a model of LPS-induced macrophages showed that inhibition of H_2_S production by gene silencing of CSE reduces inflammation using the mechanism of NF-κB downregulation by reducing ERK phosphorylation. In contrast, in an experimental design using human fibroblast-like synoviocytes, exogenous H_2_S treatment (0.05 to 5 mM) resulted in higher mRNA levels for several pro-inflammatory genes independent of NF-κB activation, and the mechanism that may rely on this is mediated by mitogen-activated protein kinase (MAPK) activation, which appears to play a role in the expression of pro-inflammatory genes [148]. 

## 6. Relationship between GSH, Garlic Derivatives and H_2_S

It is known that cysteine, GSH, and H_2_S are closely linked in metabolic processes, and several factors may contribute to the increase of GSH levels in conditions of increased levels of H_2_S [31]. H_2_S has been described as playing a role inn intracellular GSH levels by increasing the transport of cysteine, helping the redistribution of GSH into mitochondria, and contributing to the protection of cells from oxidative stress. In this study, the authors reported that H_2_S donor treatment rapidly increased the reduction of cystine into cysteine in cultured primary cortical neurons, and furthermore, cysteine is efficiently transported into cells to be used for GSH synthesis. Consequently, H_2_S enhanced the activity of GGT and GSS and increased the levels of mitochondrial GSH [32]. Additionally, enzymes that are required for the conversion of methionine into cysteine have been shown to decline over the years and have diurnal variation, which affect the synthesis of GSH, and consequently, compromise the levels of H_2_S in the body [34]. Recently, researchers have demonstrated that S-glutathionylation, a post translational modification of proteins, is a mechanism responsible for increasing CBS enzyme activity, consequently increasing H_2_S production under oxidative stress conditions [149].

As previously mentioned, sulfur-containing compounds from garlic are considered to be potential anti-inflammatory mediators that reduce the clinical conditions related to chronic diseases. Recent studies suggest that the benefits associated with garlic-derived sulfur compounds are closely linked to H_2_S production. Liang et al. [150] tested the releasing dinamic of H_2_S from the reaction between garlic oil polysulfides diallyl trisulfide (DATS) and diallyl disulfide (DADS) and GSH. The authors demonstrated that DATS releases H_2_S imediately using GSH as a thiol-disulfide exchanger both in media and in cell culture, while DADS slowly releases H_2_S in the presence of GSH, therefore, DADS may be more preferable for use to achieve physiological effects. Likewise, DeLeon et al. [151] also tested the production of H_2_S from garlic oil in buffer and in HEK cells in the presence of several different thiols. Results showed that H_2_S release from garlic oil compounds requires other low-molecular weight thiols, such as cysteine and GSH. In accordance with these two studies, Benavides et al. [90] suggested that H_2_S mediates the vasoactivity of garlic, in which they found that garlic-derived organic polysulfides cross the cell membrane and react with GSH to produce H_2_S in red blood cells, leading to vasorelaxation via K_ATP_-linked hyperpolarization in vascular smooth muscle cells. Similar results were observed by Predmore et al. [91], where mice treated with DATS showed increased levels of H_2_S in blood and myocardial tissues, which gives DATS a protective effect against ischemic myocardium. Moreover, SAC from AGE has been suggested as a cardioprotective compound by increasing the gene expression and activity of the CSE enzyme in rats with acute infarcted myocardium, consequently increasing H_2_S levels [152]. 

**Figure 1:**
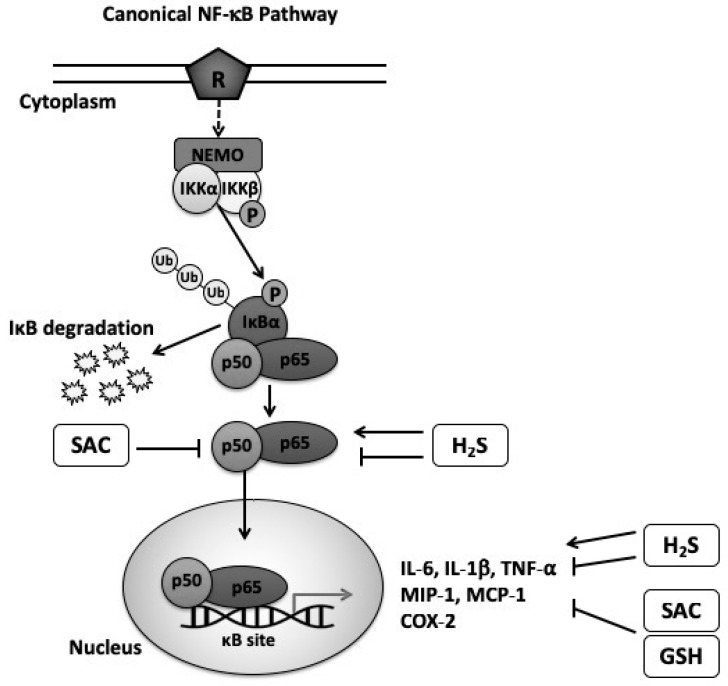
Schematic illustration of the proposed mechanism by which GSH, SAC and H_2_S affect the activation of the canonical NF-κB pathway. The canonical NF-κB pathway is the major pathway responsible for controlling inflammatory events through translocation of p50/p65 heterodimer to the nucleus. This event induces the production of pro-inflammatory cytokines and other chemochines, such as IL-6, TNF-α, IL-1β, MIP-1α, MPC-1 and COX-2 enzyme. It has been suggested that GSH has anti-inflammatory effects by reducing the levels of pro-inflammatory cytokines production. SAC, a constituent from AGE, has been shown to inhibit p50/p65 activation and the levels of pro-inflammatory cytokines. Contrarily, H_2_S seems to have both inhibitory and activating effects in the NF-κB pathway. Abbreviations: R (receptor); NF-κB (nuclear factor kappa-light-chain-enhancer of activated B cells); NEMO (NF-kB essential modulator); IκB (inhibitor of kappa B); IκBα (inhibitor of kappa B alpha); IKKα (IκB kinase alpha); IKKβ (IκB kinase beta); P (phosphorylation); Ub (ubiquitination); TNF-α (tumor necrosis factor-alpha); MIP-1 (macrophage inflammatory protein-1); MCP-1 (monocyte chemoattractant protein-1); COX2 (cyclooxygenase-2), SAC (S-allyl cysteine); GSH (glutathione), H_2_S (hydrogen sulfide).

Moreover, AGE, GSH, and H_2_S are known to affect NF-κB activation, the major pathway involved in inflammation. In fact, it has been suggested that both AGE and GSH act as anti-inflammatory mediators by attenuating NF-kB activation, however, H_2_S has been shown to work in both ways depending on the study model conditions and the concentration of the endogenous source. Figure 1 illustrates the mechanistic effect of GSH, SAC and H_2_S in the canonical NF-κB pathway. 

## 7. Conclusions

In summary, a series of studies have reported that sulfur-containing compounds play a crucial immunomodulatory role, especially in the inflammatory response, and also in the regulation of redox status and oxidative stress response. GSH, garlic derivatives and H_2_S are known to affect the activation of two important regulatory pathways: NF-kB and Nrf2, which are important components in alleviating complications related to chronic diseases and stress conditions. Despite garlic and GSH being considered as anti-inflammatory mediators, H_2_S has been shown to act in both ways depending on the experimental conditions. However, further investigations are necessary to elucidate the role of H_2_S in the regulation of inflammatory responses and how sulfur-containing compounds, such as garlic compounds and GSH, may contribute to this process.

## Figures and Tables

**Table 1:** Summary of previous studies on the effect of glutathione in the immune response.

Model	Treatment	Dose	Immune Response	Reference
Cell culture of isolated DC and T cells from study mice	Cysteine	*T Cell Proliferation Assay:* 50 μM cysteine *Metabolic Labeling:* 1 μCi/mL [^35^S] cysteine per 2 × 10^6^ cells	-Redox modeling of Tregs through reduction of GSH synthesis in DC. -Blocking of GSH redistribution from the nucleus to the cytoplasm in Tregs in response to T cell activation.	[96]
Cell culture of isolated leukocytes from healthy human blood	GSH-oxidizing agents (diamide and BHP)	50 nmoL per 10^6^ cells	-Regulation of phagocytosis in PMN through the inhibition of the assembly of microtubules and consequently reduction of H_2_O_2_ production in GSH homeostasis.	[97]
Cell culture of isolated splenic naive T cells from mice	NAC in GSH depleted cells	20 mM NAC per 10^6^ cells in the presence of 1 mM BSO	-Inhibition of T cell proliferation and reduction IL-2 production in GSH depleted cells. -Stimulation of T cell proliferation and increase IL-2 production in restored GSH cells.	[98]
Cell culture of isolated glomerular mesangial cell from rats	GSH	3–5 mM GSH per 0.5–1 × 10^6^ cells	-Inhibition of the complement activation cascade and the binding of antibodies to antigens.	[99]
Human randomized controlled clinical trial in HIV-infected patients	NAC	~7 g/day, oral	-Increase in GSH levels in the blood and in CD4 T cells.	[100]
Human randomized controlled clinical trial in HIV-infected patients	Liposomal-GSH	1.26 g/day, oral	-Increase in the levels of total GSH in CD4 T cells, IL-6, IL-12, IL-2, IFN-γ and reduction in the levels of IL-10.	[101]
Patients with cirrhosis	GSH precursor (oxathizolidine-4-carboxylic acid)	210 mg/kg/day, intravenous	-Reduction in the levels of IL-8 and IL-6 from isolated monocytes.	[102]

Abbreviations: DC (dendritic cells); GSH (glutathione); NAC (N-acetylcysteine); BSO (L-buthionine-sulfoximine); HIV (human immunodeficiency virus); IL (interleukin); IFN-γ (interferon-gamma).

**Table 2:** Previous studies on the effect of garlic derivatives in the immune response.

Model	Treatment	Dose	Immune response	Reference
Cell culture of isolated PBMC from healthy individuals	Fresh garlic extract	0.5–4 μg/mL per 10^6^ cells	-Decrease of IL-17 expression when stimulated with PHA.	[48]
Cell culture of 3T3-L1 adipocytes	Alliin	100 μmol/L (number of cells were not specified)	-Down-regulation of IL-6, MCP-1 genes expression and decrease of IL-6, MCP-1 protein levels. -Up-regulation of genes involved with immune response.	[127]
Cell culture of human colorectal cancer SW480 cells	DADS	2.5–40 μmol/L (number of cells were not specified)	-Reduction of phosphorylation and nuclear translocation of NF-κB/p65 protein in a dose dependent way.	[128]
Cell culture of isolated splenocytes from mice	AGE	0.5-1 v/v% per 0.2–1 × 10^7^ cells	-Increase in NK cell activation and cytotoxic T cells with suppression of tumor cell growth. -Increase of IL-2, TNF-α and IFN-γ levels in spleen cells.	[130]
Cell culture of Jurkat T cells	SAC	0, 0.5, 1, 1.5, or 2 mg/mL of SAC in 10^6^ cells	-Inhibition of NF-κB p50/65 heterodimer activation.	[3]
Mice with leukemia	DATS	1 and 10 mg/kg, oral	-Increase of phagocytosis activity on PBMC.	[123]
Mice with implanted breast tumors	Purified protein fraction from garlic extract	0.01–0.04 mg/mL, inoculation into the lesion	-Increase of T-cell activation and increase of intra-tumor infiltration of lymphocytes in all doses	[124]
Mice with alcoholic fatty liver disease	Allicin	5 mg/kg/day and 20 mg/kg/day, oral	-Reduction of TNF-α, IL-1β and IL-6 levels with 20 mg dose.	[46]
Mice	Purified fraction of garlic extract	10–40 mg/mL, oral	-Increase of NK activity with the 20 mg/mL dose.	[125]
Mice with colitis	Alliin	500 mg/kg, oral	-Suppression of LPS-induced AP-1/NF-κB/STAT-1 activation.	[129]
Mice exposed to Aflatoxin B_1_	AGE	20 mg/kg/day, intraperitoneal	-Decrease in IFN-γ and IL-4 levels in isolated splenocytes. -Decrease in the number of Treg in the spleen.	[131]
Mice with implanted fibrosarcoma tumor	AGE	100 mg/kg (containing 0.4 g of garlic/mL), intraperitoneal	-Increase in spleen lymphocyte subpopulation ratio and IFN-γ levels. -Increase in the amount of CD4^+^/CD8^+^ ratio.	[132]
Rats with induced neuroinflammation	AGE	125, 250, 500 mg/kg, oral	-Decrease in the density of CD11b-positive microglia immunoreactivity and decrease in up-regulation of IL-1β in hippocampal region at all doses.	[133]
Mice with diabetes	SAC	0.5 g/L, oral by drinking water	-Reduction in renal levels of IL-6 and TNF-α. -Suppression of renal NF-κB activity, mRNA expression and protein production.	[5]
Rats with diabetes	SAC	150 mg/kg/day, oral	-Reduction in hippocampal NF-κB, TLR4 and TNF-α.	[6]
Rats with chromium (VI)-induced hepatotoxicity	SAC	100 mg/kg, oral	-Decrease in the expression of NF-κB, TNF-α and iNOS in the liver.	[136]
Human randomized crossover study in healthy individuals	Raw crushed garlic	5 g, oral	-Increase of *NFAM1* gene expression.	[126]
Human randomized controlled clinical trial in patients with advanced colon, liver and pancreatic cancer	AGE	500 mg/day, oral	-Increase in NK cell count and activity.	[64]
Human randomized controlled clinical trial in healthy individuals	AGE	2.56 g/day, oral	-Increase in γδ-T cells and NK cells. -Attenuation of the severity of reported of cold and flu-like symptoms.	[134]
Human randomized controlled clinical trial in obese individuals	AGE	3.6 g/day, oral	-Increase in γδ-T cell population and decrease in NKT cell population, IL-6 and TNF-α levels in the serum.	[135]

Abbreviations: PBMC (peripheral blood mononuclear cells); MCP-1 (monocyte chemoattractant protein 1); DADS (diallyl disulfide); NF-κB (nuclear factor kappa-light-chain-enhancer of activated B cells); NF-κB/p65 (nuclear factor kappa-light-chain-enhancer of activated B cells/p65 subunit); AGE (aged garlic extract); NK (natural killer); TNF-α (tumor necrosis factor-alpha); IFN-γ (interferon-gamma); SAC (S-allyl cysteine); DATS (diallyl trisulfide); LPS (lipopolysaccharide); AP-1 (activator protein 1); STAT-1 (signal transducer and activator of transcription 1); CD (clutter of differentiation); TLR (toll-like receptor); iNOS (inducible nitric oxide synthase).

**Table 3:** Previous studies on the effect of hydrogen sulfide on inflammation.

Model	Treatment	Dose	Immune response	Reference
Cell culture of RAW264.7 cells	FW1256 (H_2_S donor)	200 μM per 2 × 10^5^ or 10^6^ cells	-Decrease in TNF-α, IL-6, PGE_2_, NO levels and expression of IL-1β, COX2, and iNOS. -Inhibition of NF-κB activation.	[14]
Cell culture of THP-1 cells	NaHS	0.01, 0.1, 0.5 or 1 mM per 2 × 10^6^ cells	-Decrease in IL-6 and TNF-α levels in a dose dependent manner.	[141]
Cell culture of U937 cells	NaHS	0.01, 0.1, or 1 mM (number of cells were not specified)	-Increase in mRNA expression and protein levels of TNF-α, IL-1β and IL-6 with 0.1 and 1mM doses. -Up-regulation of CD11b with 0.1 mM dose. -Increase in NF-κB p65 activity with 0.1 and 1mM doses.	[13]
Cell culture of human fibroblast-like synoviocytes	NaHS	0.05–5 mM (number of cells were not specified)	-Increase in mRNA expression of TNF-α, IL-8, IL-1α, IL-1β and COX-2 at 2 mM dose. -Increase in mRNA expression of IL-8 and COX-2 at doses below 0.25 mM. -NaHS did not affect NF-κB activation at none of the doses.	[148]
Mice with colitis	ATB-429 (H_2_S donor)	33–130 mg/kg, oral	-Reduction of mRNA expression of TNF-α, IFN-γ, IL-1, IL-2, IL-12 p40 and RANTES.	[142]
Rats with colitis	NaHS and Lawesson’s reagent (H_2_S donors)	30 μmol/kg, intracolonically	-Reduction of mRNA expression of TNF-α with both donors. -Reduction in TNF-α protein levels with NaHS.	[143]
Mice with LPS-induced airway inflammation	GYY4137 (H_2_S donor) and NaHS	GYY4137:0.3 or 1 mg/kg NaHS: 1 or 3 mg/kg, intranasal	-Prevention of neutrophil increase in bronchoalveolar lavage fluid with both donors. -Prevention of IL-1β levels increase with both donors.	[144]
Rats with chronic renal failure	NaHS	100 μmol/kg, intraperitoneal	-Decrease in TNF-α, IL-6, IL-10, and MCP-1 renal levels with both donors. -Decrease in NF-κB-p50, p65, and p-p65 levels with both donors.	[145]

Abbreviations: COX2 (cyclooxygenase-2); iNOS (inducible nitric oxide synthase); RANTES (regulated on activation, normal T cell expressed and secreted); NaHS (sodium hydrosulfide).
